# The role of PAX1 methylation in predicting the pathological upgrade of cervical intraepithelial neoplasia before cold knife conization

**DOI:** 10.3389/fonc.2022.1064722

**Published:** 2023-01-11

**Authors:** Mingzhu Li, Chao Zhao, Yun Zhao, Jingran Li, Jingyuan Wang, Hongxue Luo, Zhijian Tang, Yan Guo, Lihui Wei

**Affiliations:** Department of Obstetrics and Gynecology, Peking University People’s Hospital, Beijing, China

**Keywords:** cervical intraepithelial neoplasia, cold knife conization, PAX1, methylation, pathological upgrade

## Abstract

**Objective:**

To explore the ability of PAX1 methylation (PAX1^m^) to predict the pathological upgrade of cervical intraepithelial neoplasia (CIN) before cold knife conization (CKC).

**Methods:**

A total of 218 women that underwent colposcopy-directed biopsy (CDB) pathology for the confirmation of CIN2 and CIN3 between December 2020 to September 2021 were enrolled in this study. The methylation levels of PAX1 (ΔCp_PAX1_) were determined by quantitative methylation-specific polymerase chain reaction (qMSP). Receiver operating characteristic curve was used to identify the optimal cut-off value of ΔCp_PAX1_ for predicting the pathological upgrade of disease.

**Results:**

In the CDB-confirmed CIN2 group, 36% of CIN2 was found to have pathologically upgraded to CIN3 and 30% regressed to low-grade squamous intraepithelial lesion (LSIL) and below, and none of CIN2 upgraded to early-stage cervical cancer (ESCC) after CKC. In the CDB-confirmed CIN3 group, 19.5% (23/118) of CDB-confirmed CIN3 were pathologically upgraded to ESCC after CKC. Regardless of CIN2 or CIN3, the ΔCp_PAX1_ level of women with upgraded pathology after CKC was significantly lower than that of women with degraded pathology. The optimal △Cp_PAX1_ cut-off value in predicting CIN3 to be upgraded to ESCC after CKC was 6.360 and the area under the curve (AUC) was 0.814, with similar sensitivity (78.3%) and higher specificity (84.2%) than cytology≥LSIL (Se:78.3%;Sp:58.9%) and HPV16/18 positive (Se:73.9%;Sp:46.3%) patients.

**Conclusions:**

PAX1^m^ could be a promising auxiliary marker in predicting the pathological upgrade of CIN before CKC. We found that if the △Cp _PAX1_ cut-off value is lower than 6.360, it is highly suggestive of invasive cervical cancer.

## 1 Introduction

Persistent infection of high-risk human papillomavirus (hr-HPV) is an important risk factor for the development of cervical intraepithelial neoplasia (CIN) and cervical cancer. In 2020, the World Health Organization (WHO) classification of female genital tumours was updated from the original three-level classification of cervical intraepithelial neoplasia (CIN1, CIN2, CIN3) to a two-level classification that included low-grade squamous intraepithelial lesions (LSIL/CIN1) and high-grade squamous intraepithelial lesions (HSIL/CIN2 and CIN3) (1). HSIL is recognized as the true precancer with a higher risk of progression. However, it is difficult to determine whether or when a patient with HSIL will progress to invasive cervical cancer from an individual perspective, in fact, some patients may already have occult cervical cancer when diagnosed with HSIL. HSIL are primarily treated with cervical conization, including cervical cold knife conization (CKC) or loop electrode excision procedure (LEEP). On the other hand, most CIN2 lesions (60%), particularly in young women (<30 years), regress spontaneously, indicating that active surveillance, rather than immediate intervention, is justified, especially if patients adhere to monitoring (2). However, there are some limitations in the consistency between pathological assessment *via* colposcopy-directed biopsy(CDB) and final pathological diagnosis after conization, with an upgrade rate of 23.1% and degrade rate of 33.6% post-conization pathology (3).

To date, there are no accurate tests to determine whether CIN lesions have a tendency to regress or progress. The HPV genotype present in affected patients could not provide additional information to predict high-grade disease progression (4). Although the proportion of severe lesions caused by HPV16/18 has increased over time, its potential for progression remains uncertain (5, 6). Gene methylation is a kind of epigenetic modification that can contribute to the accumulation of mutated genes over time and methylation may play an important role in tumor genesis and progression. As such, using methylation as a marker due to its high sensitivity for cancer has potential as a primary screening tool. It may also be used for the management of women with CIN lesions to prevent overtreatment of CIN2/CIN3 lesions (7).

In particular, the efficacy of paired boxed gene 1 (PAX1) methylation (PAX1^m^) as a biomarker for the detection of CIN3 or worse (CIN3+) has been demonstrated in various studies (8–10). PAX1^m^ can be used as a triage method for women with atypical squamous cells of undetermined significance (ASCUS) and has shown better diagnostic performance than HPV-DNA in predicting CIN2+ (11). Besides, PAX1^m^ has a comparable clinical performance to cytology and better accuracy and specificity than HPV16/18 as a triage tool for detecting CIN3+ in women with hr-HPV (12). PAX1^m^ has also been reported to predict the efficacy of concurrent chemo-radiotherapy in cervical cancer (13), and is a potential biomarker for monitoring the prognosis of cervical adenocarcinoma (14). However, few studies have evaluated PAX1 gene methylation before conization, it has been previously reported that PAX1^m^ would be a suitable alternative method to conventional options and it has the ability to predict the outcome of conization in CIN3 cases (15). However, the role of PAX1^m^ in predicting the pathological upgrade of CIN2 is unclear. In this study, we aim to investigate the predictive value of PAX1^m^ status in determining the upgrade tendency of CIN2 and CIN3. This information would help patients and doctors make more individualized treatment decisions.

## 2 Materials and methods

### 2.1 Participants, study design, and sample collection

In total, 247 women with pathologically confirmed HSIL by CDB were included in this study at the Peking University People’s Hospital between December 2020 to September 2021. The exclusion criteria were as follows: (1) CDB revealed the presence of squamous cell cancer, adenocarcinoma *in situ* (AIS), or adenocarcinoma, (2) inability to undergo CKC, (3) inadequate DNA concentration in cell samples, (3) HSIL in patients who were also pregnant, had immune system diseases, or receiving immunosuppressive therapy, (4) patients that had a history of cervical disease treatment, hysterectomy, or chemoradiotherapy. Of the 247 women with CDB-confirmed HSIL, 16 cases of CIN2 and 4 of CIN3 chose observational follow-up rather than CKC and 9 cases were determined to have AIS. Therefore, a total of 218 women with pathologically confirmed HSIL by CDB were included in this study.

Exfoliated cervical cell samples were collected after biopsy pathology had confirmed HSIL within 7 days before the CKC procedure. Briefly, a vaginal speculum was placed to expose the cervix and cervical exfoliation was performed at the squamocolumnar junction of the cervix using a sampling brush. The sampling brush was then placed into a 20mL PreservCyt1 solution (Hologic, Marlborough Mass, USA, DOC sample) for testing. All specimens were tested for cytology, HPV detection, and PAX1 methylation. We informed patients of the research programs and obtained written consent before CKC. The study was approved by the Institutional Review Board of Peking University People’s Hospital (2020PHB298-01).

### 2.2 Quantitative methylation-specific PCR

Cervical exfoliated cells were centrifuged and stored in phosphate-buffered saline at -20°C. Genomic DNA was extracted using standard protocols and then converted to bisulfite form using the EZ DNA Methylation-Gold kits (Zymo Research, Irvine, CA, USA). Quantitative methylation-specific PCR (qMSP) was performed using a Light Cycler LC480 system (Roche Applied Science, Penzberg, Germany) to determine the methylation level of PAX1 according to the manufacturer’s instructions (Hoomya Ltd, Hunan, P.R China). Type II collagen gene (COL2A) was used as an internal reference. The △Cp is the difference between the △Cp values for PAX1 and COL2A. The methylation level (△Cp) was assessed by the following formula: △Cp=Cp target gene - Cp Col2A (16). A smaller △Cp_PAX1_ value denotes a higher degree of PAX1 methylation detected in the collected samples.

### 2.3 HPV genotyping

Type-specific HR-HPV viral genotyping was simultaneously measured using a BioPerfectus Multiplex Real-Time PCR(BMRT) assay. BMRT is a PCR-based assay for the detection of high-risk HPV strains and it was performed using a fluorescence-based multiplex HPV DNA genotyping kit (Bioperfectus Ltd, Jiangsu, P.R. China). This assay can detect 14 high-risk HPV subtypes (HPV 16, 18, 31, 33, 35, 39, 45, 51, 52, 56, 58, 59, 66, and 68) and 7 medium- and low-risk subtypes. For this study, all types specifically refer to high-risk HPV.

### 2.4 Pathological diagnosis of upgraded disease after conization

Colposcopic impressions were made according to the American Society for Colposcopy and Cervical Pathology (ASCCP) standard, multiple biopsies targeting all areas with acetowhitening, metaplasia, or higher abnormalities are recommended. At least 2-4 targeted biopsies from distinct acetowhite lesions should be taken (17). A circular knife cut is made 3 mm peripheral to the abnormal transformation zone (ATZ). The knife is angled toward the endocervical canal and cuts deeper into the stroma, the depth of excision depends on the type of TZ according to 2011 Colposcopic Terminology of International Federation for Cervical Pathology and Colposcopy (IFCPC) (18). Cervical biopsy and CKC specimens were histologically examined and classified according to the 2020 WHO classification of female genital tumours (1), which reported as HSIL(CIN2) and HSIL (CIN3), p16 immunohistochemistry was used only in morphologically ambiguous cases when HSIL is suspected according to the guidance provided by the Lower Anogenital Squamous Terminology (LAST) Project (19). The highest pathological grade was taken as the final pathological diagnosis. Pathological upgrade of disease is defined as CIN2_CDB_ (CDB-confirmed CIN2) →CIN3_CKC_ (CKC-confirmed CIN3) and pathology-confirmed CIN3_CDB_ (CDB-confirmed CIN3) →ESCC_CKC_ (CKC-confirmed early-stage cervical cancer). The cervical lesions were diagnosed by two professional pathologists.

### 2.5 Statistical analysis

The samples were characterized using descriptive statistics in two groups of biopsy diagnosis. The Mann-Whitney test was utilized to analyze the differences between △Cp_PAX1_ levels. We used restricted cubic spline models fitted for logistic odds ratio with 3 knots of PAX1^m^ using statistical software (rms in R, version 4.1.2) (**Supplementary Figure 1**). We divided the △Cp_PAX1_ into 3 groups to form grade variables and assigned the group names of Low (1:△Cp >15), Moderate (2: 9 ≤ △Cp ≤ 15), and High (3:△Cp ≤ 9). Logistic regression was used to evaluate the odds ratio (OR) and control for confounding factors (e.g., HPV, cytology) (model 1). The receiver operating characteristic (ROC) curve was used to identify the optimal cut-off value of PAX1^m^ for predicting pathological upgrade of disease. Sensitivity (Se), specificity (Sp), positive predictive value (PPV), and negative predictive value (NPV) were calculated. Confidence intervals for Se and Sp are Clopper-Pearson confidence intervals. Confidence intervals for the predictive values are the standard logit confidence intervals (20). SPSS software version (Version 26.0, SPSS, Inc, Chicago, IL) were used for statistical analysis. All differences were considered two-sided and statistically significant at *P* < 0.05.

## 3 Results

**Figure 1:**
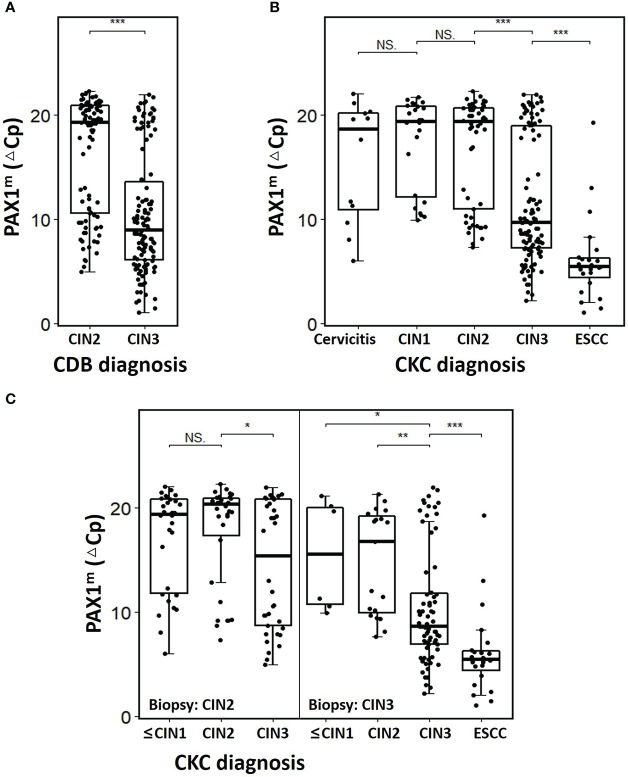
The PAX1^m^ distribution of cervical lesions diagnosed by CDB and CKC. **(A)** Comparison of ΔCp_PAX1_ levels of CIN2 and CIN3 diagnosis by CDB; **(B)** Comparison of ΔCp_PAX1_ levels of different cervical lesions diagnosed by CKC; **(C)** Comparison of ΔCp_PAX1_ levels changing in upgraded, maintained, or degraded lesions after CKC. The middle line is the median; the box shows the inter-quartile range (IQR), and the whiskers extend to, at most, 1.5 times the IQR. *p<0.05, **p<0.01, ***p<0.001, NS: not significant.

Among the 218 women with CDB-confirmed HSIL, 100 cases were CIN2_CDB_ and 118 cases were CIN3_CDB_. The mean age was 40.7 ± 10.8 years (22-69). The median △Cp_PAX1_ was 19.3 (10.5-20.9) and 8.9 (6.0-14.0) in CIN2_CBD_ and CIN3 _CBD,_ respectively, which was significantly different (**Figure 1A**). In the CIN2_CBD_ group, 36% of CIN2 was pathologically upgraded to CIN3 and 30% regressed to LSIL and below, none of the CIN2 cases upgraded to SCC after CKC, and the positive margin rate was only 2%. However, in the CIN3_CBD_ group, 19.5% (23/118) of CDB-confirmed CIN3 were pathologically upgraded to ESCC after CKC. The detailed characteristics are presented in **Table 1**.

**Table 1:** Characteristics of CDB pathology-confirmed CIN2 and CIN3.

Characteristics	CIN2 _CBD_ (n = 100)			CIN3 _CBD_ (n = 118)		
	Frequency		Percent	Frequency		Percent
**Age median (IQR)**	40 (35.2-50.0)			40 (35.5-51.0)		
Cytology						
LSIL-^*^	79		79.0	69		58.5
ASC-H+^^^	21		21.0	49		41.5
HR-HPV genotype						
HPV16/18(+)	34		34.0	68		57.6
Other 12 HR-HPV(+)	59		59.0	44		37.3
Negative	7		7.0	6		5.1
CKC pathology						
Cervicitis	8	8.0		4	3.4	
CIN1	22	22.0		2	1.7	
CIN2	34	34.0		19	16.1	
CIN3	36	36.0		70	59.3	
ESCC	–	–		23	19.5	
Margin status						
Negative	98	98.0		90	76.3	
Positive	2	2.0		28	23.7	

^*^including negative for intraepithelial lesion or malignancy (NILM), atypical squamous cells of undetermined significance (ASCUS), and low-grade squamous intraepithelial lesion (LSIL); ^^^including: atypical squamous cells cannot exclude HSIL(ASC-H),atypical glandular cell(AGC), and high-grade squamous intraepithelial lesion(HSIL). CKC, cold knife conization; CIN, cervical intraepithelial neoplasia; ESCC, early-stage cervical cancer; IQR, inter quartile range.

After conization, the△Cp_PAX1_ level of ESCC_CKC_ was significantly lower than that of CIN3_CKC_, and that of CIN3_CKC_ was lower than CIN2_CKC_, with statistically significant differences (*p*<0.001) (**Figure 1B**). Within the CIN2_CDB_ group, there was no difference in △Cp_PAX1_ between CIN2_CDB_ that had degraded to ≤CIN1_CKC_ and those that had maintained at CIN2_CKC_, but there was a significant difference between those that had maintained at CIN2_CKC_ and those that upgraded to CIN3_CKC_. Among the CIN3_CDB_ group, △Cp_PAX1_levels were significantly lower in those that had upgraded to ESCC_CKC_ than those that had maintained at CIN3_CKC_ and downgraded to ≤CIN2_CKC_ (*p*<0.001) (**Figure 1C**). Regardless of CIN2 or CIN3 status, △Cp_PAX1_ level of women with upgraded pathology after CKC was significantly lower than that of women with degraded pathology (detailed information see **Supplementary Table 1**).

When analyzing PAX1^m^ at different thresholds, it was found that the risk of CIN3+ increased significantly when ΔCp_PAX1_ < 7, while the trend turns flat when ΔCp_PAX1_ > 15 (**Supplementary Figure 1**). We divided the different threshold levels of PAX1^m^ according to high-, medium-, and low-risk for CIN2+, CIN3+, and ESCC. When ΔCp_PAX1_ was 6.4 (1.1-9.0), the OR values of CIN2+, CIN3+, and ESCC were 12.52 (2.85-55.00), 20.61 (8.08-52.57), and 34.07 (4.45-261.08), respectively. In order to adjust variables that would have effects on PAX1^m^, we established PAX1^m^_Model 1_(shown in the “Methods” section), and further confirmed that the risk of ESCC, CIN3+, and CIN2+ was still high, and the OR values were 24.85 (3.17-194.67), 19.27 (7.39-50.22), and 11.98 (2.68-53.65), respectively (**Table 2**), indicating that if ΔCp_PAX1_ less than 6.4, it should be alert for the occurrence of high-grade lesions or even cervical cancer.

**Table 2:** PAX1^m^ levels stratified by low-, medium-, and high-risks of CIN2+, CIN3+ and ESCC.

Variable	OR/Adjusted OR (95%CI) ^a^			P for trend ^b^
PAX1 ^m^	Low N = 93	Moderate N = 51	High N = 74	
Median (range)	20.4 (16.3-22.3)	10.6 (9.11-14.35)	6.4 (1.1-9.0)	
OR, CIN2+	1.0	1.43 (0.62-3.28)	12.52 (2.85-55.00)	<0.001
*P*		0.404	0.001	
OR, CIN3+	1.0	2.21 (1.10-4.44)	20.61 (8.08-52.57)	<0.001
*P*		0.025	<0.001	
OR, ESCC	1.0	3.76 (0.33-42.46)	34.07 (4.45-261.08)	<0.001
*P*		0.285	0.001	
PAX1^m^_Model 1_^c^				
OR, CIN2+	1.0	1.36 (0.58-3.18)	11.98 (2.68-53.65)	<0.001
*P*		0.473	0.001	
OR, CIN3+	1.0	2.00 (0.98-4.08)	19.27 (7.39-50.22)	<0.001
*P*		0.056	<0.001	
OR, ESCC	1.0	3.20 (0.27-37.08)	24.85 (3.17-194.67)	<0.001
*P*		0.353	0.002	

^a^ ORs and 95%CI were calculated with the use of the logistic regression.

^b^ P for trend, from a 1 degree-of-freedom trend test.

^c^ The following variables were included to control for the effects of PAX1^m^: hrHPV [other hrHPV (+) except HPV16/18, HPV16/18 (+)] and cytology (<LSIL, LSIL+).

**Figure 2:**
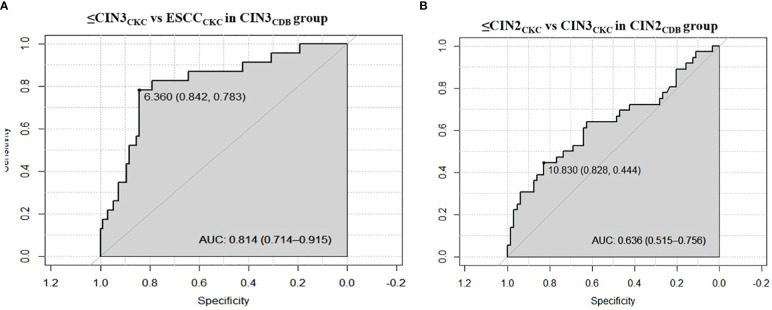
Receiver operating characteristic curves of performance of PAX1 methylation. **(A)** The optimal ΔCp_PAX1_cut-off value in predicting whether CIN3 would be upgraded to ESCC after CKC; **(B)** The optimal ΔCp_PAX1_cut-off value in predicting whether CIN2 would be upgraded to CIN3.

The optimal ΔCp_PAX1_ cut-off value in predicting whether CIN3 would upgrade to ESCC after CKC was 6.360 and the area under the curve (AUC) was 0.814 (95% CI: 0.714–0.915), with similar sensitivity (78.3%) but higher specificity (84.2%) than cytology≥ LSIL (Se:78.3%;Sp:58.9%) and HPV16/18 positive (Se:73.9%;Sp:46.3%). The optimal ΔCp_PAX1_ cut-off value in predicting whether CIN2 would upgrade to CIN3 was 10.830 and the AUC was 0.636 (95% CI: 0.515–0.756), with lower sensitivity (44.4%) but higher specificity (82.8%), compared with cytology>ASCUS (Se:44.4%;Sp:45.3%) and HPV positive (Se:97.2%;Sp:9.4%) (**Figure 2** and **Table 3**).

**Table 3:** The Performance of ΔCp_PAX1_ in predicting CIN2 upgrade to CIN3, and CIN3 upgrade to ESCC after conization.

Test	Sensitivity % (95%CI)	Specificity % (95%CI)	PPV % (95%CI)	NPV % (95%CI)	OR (95%CI)
CIN2_CDB_ (n = 100)					
CIN3+_CKC_ (n = 36)					
△Cp_PAX1_ ≤ 10.83	44.4 (27.9-61.9)	82.8 (71.3-91.1)	59.3 (43.2-73.6)	72.6 (65.9-78.4)	3.86 (1.53-9.71)
Cytology (ASCUS+)	44.4 (27.7-61.9)	45.3 (32.8-58.3)	31.4 (22.9-41.2)	59.2 (49.3-68.3)	0.66 (0.29-1.51)
hrHPV (+)	97.2 (85.5-99.9)	9.4 (3.5-19.3)	37.6 (35.4-39.9)	85.7 (42.9-98.0)	3.62 (0.42-31.34)
CIN3_CDB_ (n = 118)					
ESCC_CKC_ (n = 23)					
△Cp_PAX1_ ≤ 6.36	78.3 (56.3-92.6)	84.2 (75.3-90.9)	54.5 (41.8-66.7)	94.1 (88.0-97.2)	19.20 (6.18-59.67)
Cytology ≥ LSIL	78.3 (56.3-92.5)	58.9 (48.4-68.9)	31.6 (25.0-38.9)	91.8 (83.5-96.1)	5.17 (1.77-15.10)
HPV16/18 (+)	73.9 (51.6-89.8)	46.3 (36.0-56.8)	25.0 (19.7-31.2)	88.0 (78.1-93.8)	2.44 (0.89-6.74)

Estimated sensitivity and specificity of PAX1^m^ at maximum value of Youden index.

## 4 Discussion

In our study, CDB-confirmed CIN2 and CIN3 were stratified to predict pathological progression after CKC. We found that 19.5% (23/118) of the CDB-confirmed CIN3 cases were pathologically upgraded to ESCC after CKC, which was higher than previously reported (15), indicating that CDB alone is insufficient for the diagnosis of microinvasive cervical cancer (21). In the CDB-confirmed CIN2 group, 36% of the CIN2 cases were pathologically upgraded to CIN3 and 30% regressed to LSIL and below, however, none of the CIN2 cases were pathologically upgraded to SCC after CKC. Using accurate tests to determine whether CIN lesions have a tendency to regress or progress is crucial for subsequent disease management.

Prognostic testing for CIN could dramatically alter the treatment algorithm. Underdiagnosis leads to multiple follow-up visits, and either delayed or progressed the lesion, exacerbating the potential harm to patients. Alternatively, overdiagnosis can result in unnecessary or premature treatment, especially in younger women, as inappropriate treatment significantly increases the risk of adverse outcomes in subsequent pregnancies (22). The majority of HSILs require surgery for the purpose of completely removing lesions, as well as prevent cancer, a small number of special conditions or periods (such as young or pregnant women) can be given a short-term close follow-up (23). As recommended by the ASCCP2019 guidelines, CIN2 and CIN3 should be managed separately, and for patients with CIN 2 that are more concerned about the effects of treatment on a future pregnancy outweigh their concerns about cancer, observation without treatment is acceptable (24).

However, CIN2 and CIN3 diagnosed by CDB have limitations to some extent. For example, the diagnosis of CIN2 has historically been a gray area in pathology and it is difficult for pathologists to reproduce which might be overcalled CIN1 or under-called CIN3 (25). Some pathologists even use “CIN1–2 or CIN2–3” to equivocate the classification. Based on difficulties associated with receiving an accurate diagnosis, it is challenging to determine whether or when a patient with CIN3 will progress to invasive cervical cancer from an individual perspective. In fact, some patients may already have occult cervical cancer when diagnosed with HSIL. The rate of progression to invasive cancer after conization have been reported to be about 0.3%-15% (26–28). In our study, although none of the CIN2 cases progressed to invasive cancer, 36% of the women within the afore mentioned group did progress to CIN3, and progression to invasive cancer in the CIN3 group was as high as 20%. Relying on HPV testing alone cannot accurately predict the progression of cancer satisfactorily. A better prognostic risk evaluation for CIN2 and CIN3 is needed. The integration of molecular markers in cervical cancer screening, such as DNA methylation, might help avoid unnecessary referrals and repeatedly performing diagnostic procedures, which is a waste medical resources and generate needless worry for the patient and her family (29).

The PAX1 gene is located on chromosome 20p11 and consists of a paired domain (PD) and an octapeptide domain (OP). The expression PAX1 is associated with embryogenesis, especially the development of the skeleton, thymus, and the parathyroid glands (30, 31). In 2008, Lai et al. first reported that abnormal methylation of PAX1 was associated with cervical cancer, and that the PAX1 gene was found to be silenced by hyper-methylation and under-expressed in cervical cancer biopsies (8). PAX1 can regulate cell division and differentiation, and methylation and silencing of PAX1 is closely related to the progression of precancerous lesions into cervical cancer (32). It has been reported that the disruption between kinases and phosphatases caused by PAX1 methylation is involved in cervical carcinogenesis (33). An increasing number of studies have confirmed PAX1 methylation as a promising biomarker for cervical cancer based on its ability to discriminate between high‐grade cervical lesions and normal tissues, resulting in a reduced necessity for colposcopy referral and biopsy (9, 10, 34). The current study demonstrated that the ΔCp_PAX1_ level of CIN3 determined by CDB was lower than that of CIN2 and the ΔCp_PAX1_ level of ESCC was lower than that of both CIN2 and CIN3. Regardless of CIN2 or CIN3 status, the ΔCp_PAX1_ level of women with upgraded pathology after conization was significantly lower than that of women with degraded pathology. We further stratified the PAX1^m^ level by different thresholds and found that the risk of CIN3+ increased significantly when ΔCp_PAX1_ < 7. The optimal ΔCp_PAX1_ cut-off value in predicting whether CIN3 would be upgraded to ESCC, and whether CIN2 would be upgraded to CIN3 after CKC was 6.360 and 10.830, respectively, and is more specific than using with cytology or HPV abnormalities. This concept implies that if biopsy pathology indicates CIN2 status with a ΔCp_PAX1_ less than 10.83, then the actual pathology is more likely to be upgraded to CIN3, so it is necessary to be more careful if observation without treatment was selected. If biopsy pathology indicates CIN3 with a ΔCp_PAX1_ less than 6.36, then cervical conization is inevitable because of the increased risk of pathological upgrade to early-stage cervical cancer. On the other hand, women with negative PAX1 methylation do not need immediate colposcopy or conization because of there being a relatively low short-term progression risk for cancer. For young women with CIN2 who have fertility requirements, this approach seems to be particularly important, since only hypermethylated lesions require treatment and the risk of preterm abortion due to treatment could be reduced.

## Limitation

This study has several limitations. First, since our research has not reached the follow-up endpoint, residual and recurrence of lesions have not been discussed here, and continued follow-up is needed in the future. Secondly, the sample size was not large enough and a larger longitudinal study is necessary to validate the natural history of CIN2 and CIN3 progression in relation to DNA methylation. Thirdly, only hospitalized patients with CKC were included in this study who can be followed up well, however, those patients for LEEP from outpatient were not included due to unstable follow-up. In addition, further studies are needed to explore PAX1^m^ levels after treatment and to compare PAX1^m^ changes before and after CKC. At last, further studies are also needed to determine the ideal interval of monitoring using PAX1^m^ to avoid underdiagnosis and overdiagnosis.

## Conclusions

In this exploratory study, we found that PAX1 methylation could be a promising auxiliary marker in the prediction of pathological upgrade risk in patients with CIN2 or CIN3 before conization, especially if △Cp_PAX1_ cut-off value is lower than 6.360, as we found this to be highly suggestive of invasive cervical cancer. Using PAX1 methylation as a monitoring tool could help prevent inappropriate conservative observation or ablation therapy. Further validation and prospective clinical trials are needed to confirm these findings in the future.

## Data availability statement

The raw data supporting the conclusions of this article will be made available by the authors, without undue reservation.

## Ethics statement

The studies involving human participants were reviewed and approved by Peking University People’s Hospital Ethics Committee. The patients/participants provided their written informed consent to participate in this study.

## Author contributions

Data collection, study conception, and study design was performed by ML. Personnel organization, data collection, and the cold knife conization procedure was performed by CZ. Sample collection was performed by YZ, JL, HL, ZT, JW and YG. Supervision of the research program and manuscript review and guidance during the study was provided by LW. The first draft of the manuscript was written by ML and all authors commented on previous versions of the manuscript. All authors read and approved the final manuscript.

## Supplementary material

Supplementary Figure 1 Restricted cubic splines of odds ratio for CIN3+CKC according to ΔCp_PAX1_ levels in all participants.
