# Supplementary figures and images for: The role of PAX1 methylation in predicting the pathological upgrade of cervical intraepithelial neoplasia before cold knife conization
Source: Front Oncol. 2023 Jan 11;12:1064722. doi: 10.3389/fonc.2022.1064722 (PMC9875021; doi:10.3389/fonc.2022.1064722)

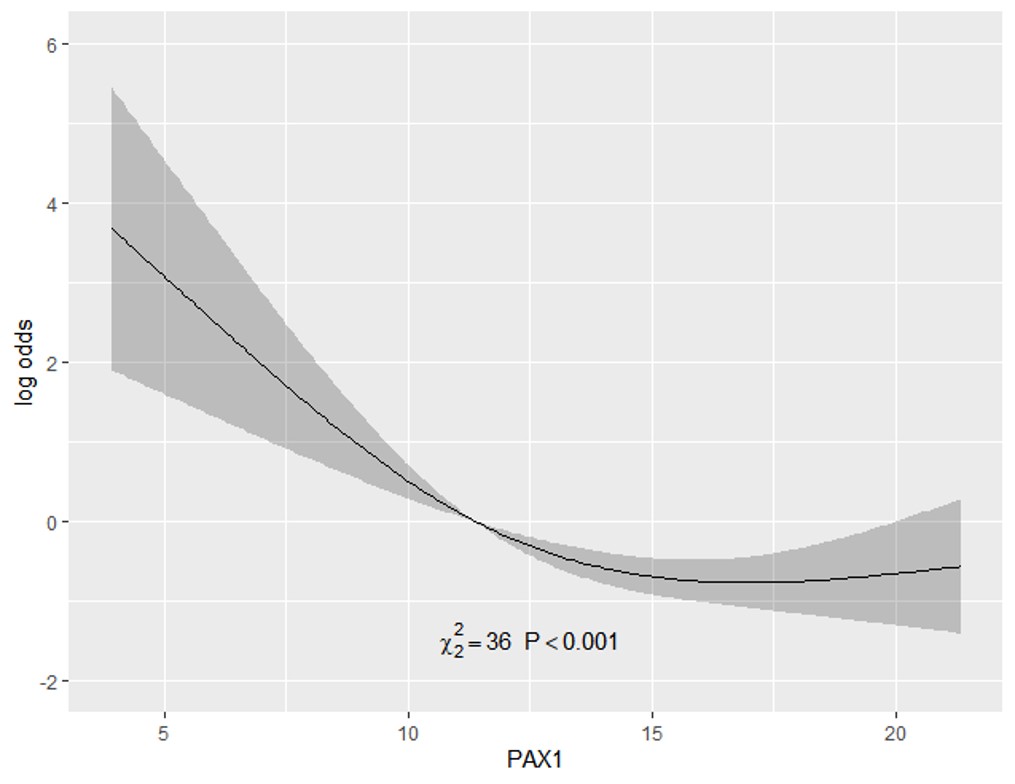

Supplement: Supplementary Figure 1 — Restricted cubic splines of odds ratio for CIN3+CKC according to ΔCpPAX1 levels in all participants. [file Image_1.jpeg]
