# Supplementary material for: The role of PAX1 methylation in predicting the pathological upgrade of cervical intraepithelial neoplasia before cold knife conization
Source: Front Oncol. 2023 Jan 11;12:1064722. doi: 10.3389/fonc.2022.1064722 (PMC9875021; doi:10.3389/fonc.2022.1064722)
Supplement: Supplementary file 2 [file Table_1.docx]

**Supplementary Table 1.** PAX1 methylation(ΔCp_PAX1_) distributions by CDB and CKC

| **Parameter** | **n** | **Median ΔCp_PAX1_ (IQR)** | ***P*** |
| --- | --- | --- | --- |
| NILM_CKC_ | 12 | 18.6(10.1-20.3) | Stepwise |
| CIN1_CKC_ | 24 | 19.4(11.8-20.9) | 0.456 |
| CIN2_CKC_ | 53 | 19.3(10.6-20.7) | 0.502 |
| CIN3_CKC_ | 106 | 9.7(7.2-19.0) | **<0.001** |
| ESCC_CKC_ | 23 | 5.4(3.9-6.3) | **<0.001** |
|  |  |  |  |
| CIN2_CDB_ | 100 | 19.3(10.5-20.9) |  |
| CIN2_CDB_→≤CIN2_CKC_ | 30 | 19.4(11.7-20.9) | 0.472^a^ |
| CIN2_CDB_→CIN2_CKC_ | 34 | 20.3(15.9-20.9) | Ref. |
| CIN2_CDB_→CIN3_CKC_ | 36 | 15.4(8.5-20.9) | **0.028**^a^ |
| CIN3_CDB_ | 118 | 8.9(6.0-14.0) | **<0.001**^b^ |
| CIN3_CDB_→≤CIN1_CKC_ | 6 | 15.5(10.4-20.4) | **0.024**^c^ |
| CIN3_CDB_→CIN2_CKC_ | 19 | 16.8(9.7-19.4) | **0.002**^c^ |
| CIN3_CDB_→CIN3_CKC_ | 70 | 8.6(6.8-11.9) | Ref. |
| CIN3_CDB_→ESCC_CKC_ | 23 | 5.4(3.9-6.3) | **<0.001**^c^ |
|  |  |  |  |
| ≤CIN2_CKC_ | 89 | 19.3(11.2-20.7) | Ref. |
| ≤CIN3+_CKC_ | 129 | 8.6(6.1-17.4) | **<0.001**^d^ |
|  |  |  |  |
| ≤CIN1_CKC_ | 36 | 19.4(11.4-20.8) | Ref. |
| CIN2+_CKC_ | 182 | 10.2(7.2-19.7) | **<0.001**^e^ |

^a^ *P* values represent comparison of the medians to CIN2_CDB_→CIN2_CKC_

^b^ *P* value between CIN2_CDB_ and CIN3_CDB_.

^c^ *P* values represent comparison of the medians to CIN3_CDB_→CIN3_CKC_.

^d^ *P* value between≤CIN2_CKC_ and CIN3+_CKC_.

^e^ *P* value between≤CIN1_CKC_ and CIN2+_CKC_.

CKC: cold knife conization; NILM: negative for intraepithelial lesion or malignancy; CIN: cervical intraepithelial neoplasia; ESCC: early-stage cervical cancer; IQR: inter quartile range; →: the pathologic diagnosis of CDB changed to diagnosis of CKC.
